# Improving Numerical Modeling Accuracy for Fiber Orientation and Mechanical Properties of Injection Molded Glass Fiber Reinforced Thermoplastics

**DOI:** 10.3390/ma15134720

**Published:** 2022-07-05

**Authors:** Riccardo Ivan, Marco Sorgato, Filippo Zanini, Giovanni Lucchetta

**Affiliations:** 1 Smart Mold s.r.l., Spin-off of the University of Padova, Viale dell’Artigianato, 42, 35013 Cittadella, Italy; riccardo.ivan@smart-mold.it; 2 Department of Industrial Engineering, University of Padova, Via Gradenigo, 6/A, 35131 Padova, Italy; marco.sorgato@unipd.it; 3 Department of Management Engineering, University of Padova, Str. S. Nicola, 3, 36100 Vicenza, Italy; filippo.zanini@unipd.it

**Keywords:** modeling, fibers, orientation, injection molding, mechanical properties

## Abstract

Local fiber alignment in fiber-reinforced thermoplastics is governed by complex flows during the molding process. As fiber-induced material anisotropy leads to non-homogeneous effective mechanical properties, accurate prediction of the final orientation state is critical for integrated structural simulations of these composites. In this work, a data-driven inverse modeling approach is proposed to improve the physics-based structural simulation of short glass fiber reinforced thermoplastics. The approach is divided into two steps: (1) optimization of the fiber orientation distribution (FOD) predicted by the Reduce Strain Closure (RSC) model, and (2) identification of the composite’s mechanical properties used in the Ramberg–Osgood (RO) multiscale structural model. In both steps, the identification of the model’s parameters was carried out using a Genetic Algorithm. Artificial Neural Networks were used as a machine learning-based surrogate model to approximate the simulation results locally and reduce the computational time. X-ray micro-computed tomography and tensile tests were used to acquire the FOD and mechanical data, respectively. The optimized parameters were then used to simulate a tensile test for a specimen injection molded in a dumbbell-shaped cavity selected as a case study for validation. The FOD prediction error was reduced by 51% using the RSC optimized coefficients if compared with the default coefficients of the RSC model. The proposed data-driven approach, which calculates both the RSC coefficients and the RO parameters by inverse modeling from experimental data, allowed improvement in the prediction accuracy by 43% for the elastic modulus and 59% for the tensile strength, compared with the non-optimized analysis.

## 1. Introduction

In the last years, the use of fiber-reinforced thermoplastics has substantially increased. Their high specific mechanical properties (elastic modulus, strength, and impact resistance) make them suitable for structural applications [1]. They are used in several engineering fields, particularly in the automotive, appliance, and power tool industries. Furthermore, they can be processed by injection molding, which enables the manufacturing of complex shapes with reduced costs for high-volume production.

The mechanical properties of such composites are highly dependent on the local morphology, which includes fiber residual length and orientation, and defects due to the process, such as weldlines and porosity [2]. In particular, fiber orientation induces a significant material anisotropy strongly influencing the mechanical behavior of molded parts. For this reason, the part and mold design phases are crucial in determining the final orientation of the reinforcement, which translates into local mechanical properties.

Fiber orientation cannot be freely varied or controlled because it depends on the gate location, part shape, and process parameters. Furthermore, fiber orientation distribution (FOD) is not uniform across the part thickness. Due to the fountain flow, fibers are well aligned with the flow direction in the skin and shear layers, but they have a mostly random distribution in the core. For all these reasons, the prediction of FOD in a complex injection molded part is still challenging.

In 1922 Jeffery described the orientation of particles immersed in a viscous fluid for the first time [3]. An advanced fiber orientation model based on Jeffrey’s equation was developed by Advani and Tucker and implemented in commercial software to account for the rotary diffusion effect [4]. Nowadays, the state-of-art short fiber orientation prediction model is the Reduce Strain Closure (RSC) model proposed by Wang and coworkers [5,6]. In this extension of the Advani and Tucker model, a scalar factor is introduced to reduce the rate of eigenvalues of the orientation tensor while the rate of eigenvectors remains unchanged [7]. The RSC model includes two additional coefficients that need to be optimized to obtain proper FOD predictions by comparing the experimental results with numerical ones. 

To predict the orientation tensor for short glass fibers, the RSC model is implemented in the most used simulation software tools, such as Autodesk Moldflow Insight, Moldex3D, and Sigmasoft. However, the accuracy of FOD predictions is highly dependent on how the RSC model coefficients are identified. 

The RSC coefficients are usually identified through an experiment in which the steady-state orientation of reinforced polymers under simple shear can be measured. Eberle and coworkers measured the FOD evolution for a polybutylene terephthalate with 30% glass fibers in a cone and plate rheometer [8]. The transient fiber orientation evolution for a polyamide 6 with 30% glass fibers was measured by Perumal and coworkers in a parallel plate rheometer using X-ray micro-computed tomography (μCT) [9]. Recently, Kugler et al. combined a sliding plate experiment with a Couette experiment to define an experimental validation curve [6]. This novel approach allowed them to cover high strains and control the initial fiber orientation, which significantly influences its evolution [10]. Using two validation cases, they showed that the identified coefficients provided accurate results in parts where the shear flow is dominant but could not predict fiber orientation in more complex flow regimes.

Adopting an alternative approach, Morak and coworkers proposed identifying the RSC model’s coefficients by inverse modeling, i.e., adapting them until the numerical simulation results fit the experimental μCT results [11]. They used the Frobenius norm to calculate the deviation between the orientation tensor evaluated from μCT scans and the orientation tensor predicted by the numerical simulation. The FOD prediction error was reduced by 33% compared with the simulations based on the default RSC coefficients, i.e., those determined through a steady-state orientation experiment under simple shear. However, they did not provide insight into how much the local mechanical properties prediction can be improved. Furthermore, to minimize the error in the Frobenius norm, they used the Response Surface Methodology (RSM) and a gradient-based algorithm, whose performance is limited by the ‘local optima’ problem and is dependent on the initial values of coefficients. Li and Luyé proposed a similar approach to capture the anisotropic rheological behavior of fiber-reinforced thermoplastics [12]. They managed to reduce the FOD prediction error by 22%, but they did not translate the fiber orientation tensor into local mechanical properties.

In this work, the identification of the RSC model coefficients was carried out using a Genetic Algorithm (GA), which can escape local optima through mutation. In order to reduce the computational time, Artificial Neural Networks (ANN) were used as a machine learning-based surrogate model to approximate the simulation results locally. Injection molding simulations and μCT scans were used to identify and compare the FOD at different locations of an injection-molded plate made of polyphenylene sulfide (PPS). The optimized FOD predictions were then translated into local mechanical properties, using tensile specimens cut out from the plate at different angles. The optimized coefficients were then used to simulate a tensile test for a specimen injection molded in a dumbbell-shaped cavity. Numerical and experimental results (obtained from both μCT scans and mechanical characterization of the injection-molded specimens) were eventually compared to assess the impact of the proposed data-driven approach on the accuracy of the mechanical behavior prediction.

## 2. Theoretical Background

### 2.1. Fiber Orientation Models

The first fiber orientation model was conceived by Jeffery, who studied the motion of ellipsoidal particles immersed in a viscous fluid [3]. The shape of the particles is included in the constant *ξ* defined as follows:

(1) $$\xi =\frac{r_{c}^{2}-1}{r_{c}^{2}+1},$$

in which *r_c_* is the aspect ratio of the particles. Jeffery considered a Newtonian fluid, linear velocity field far away from the particle, and negligible buoyancy and inertia to solve this problem. Thus, Jeffery’s equation written in vector form is:

(2) $$\frac{dp}{dt}=W\cdot p+\xi \left[D\cdot p-\left(p\cdot D\cdot p\right)p\right],$$

where ***W*** is the vorticity tensor, ***D*** is the deformation tensor, and ***p*** is the unit vector, which describes the particle’s orientation. In spherical coordinates, it is defined as follows:

(3) $$p=\left(\begin{array}{c} p_{1}\\ p_{2}\\ p_{3} \end{array}\right)=\left(\begin{array}{c} \cos\phi \sin\theta \\ \sin\theta \cos\phi \\ \cos\theta \end{array}\right),$$

In concentrated suspensions, the fibers’ mutual interaction cannot be neglected. Folgar and Tucker introduced a statistical approach by defining the distribution function $\psi_{\phi }$ as the probability of any fiber having an orientation between $\phi_{1}$ and $\phi_{2}$, reference [7]:

(4) $$P\left(\phi \right)=\int_{\phi_{1}}^{\phi_{2}}\psi_{\phi }\left(\phi \prime \right)d\phi \prime ,$$

They assumed that interactions occur whenever the center of one fiber passes within a distance *l*, equal to the fiber length, from another fiber. In this case, it can be demonstrated that the interaction is proportional to the strain rate $\dot{\gamma }$. Therefore, Folgar and Tucker modified Jeffery’s equation to include a fiber–fiber interaction coefficient, $C_{I}$, which is dimensionless and needs to be determined experimentally:

(5) $$\psi \frac{dp}{dt}=-\left(C_{I}\dot{\gamma }\right)\frac{\partial \psi }{\partial p}+\psi {\left(\frac{dp}{dt}\right)}_{Jeffery},$$

The larger the factor $C_{I}$, the more pronounced the fiber–fiber interactions. Advani and Tucker proposed to describe the fiber orientation with the tensorial notation [4]. The components of the fiber orientation tensor, ***A***, are defined as:

(6) $$a_{ij}=\oint p_{i}p_{j}\psi \left(p\right)\;dp,$$

where $tr\left(A\right)=1$. In the tensorial notation, the Folgar and Tucker model (Equation (5)) turns into the Advani and Tucker equation:

(7) $$\dot{A}=\left(W\cdot A-A\cdot W\right)+\xi \left(D\cdot A-A\cdot D-2\hat{A}:D\right)+2C_{I}\dot{\gamma }\left(I-3A\right),$$

where $\hat{A}$ is the fourth-order orientation tensor, and its components are:

(8) $$a_{ijkl}=\int p_{i}p_{j}p_{k}p_{l}\psi \left(p\right)dp,$$

Cintra and Tucker proposed to use a closure approximation of the fourth-order orientation tensor $\hat{A}=f\left(A\right)$ [13]. Advani and Tucker’s model agrees with the experimental data only for high strain values. In the actual process, fiber orientation develops more slowly, as fibers experience local strain that is lower than average because resin-rich “slip layers” absorb most of the strain. For this reason, Wang and coworkers introduced the RSC model, reference [5]:

(9) $${\dot{A}}^{RSC}=W\cdot A-A\cdot W+\xi \left\{D\cdot A+A\cdot D-2\left[\hat{A}+\left(1-k\right)\left(\hat{L}-\hat{M}:\hat{A}\right)\right]:D\right\}+2_{\kappa }C_{I}\dot{\gamma }\left(I-3A\right),$$

where the component of the fourth-order tensor $\hat{L}$ and $\hat{M}$ are given by:

(10) $$\hat{L}=\sum_{i=1}^{3}\lambda_{i}e_{i}e_{i}e_{i}e_{i},$$

(11) $$\hat{M}=\sum_{i=1}^{3}e_{i}e_{i}e_{i}e_{i}$$

where $\lambda_{i}$ and $e_{i}$ are the eigenvalues and the eigenvectors, respectively, of the second-order orientation tensor ***A***. The RSC model additionally uses a scalar factor κ<1 to slow orientation dynamics. This reduces the growth rates of the eigenvalues by a constant factor but does not affect the rotation rate of the eigenvectors. The smaller the factor κ, the slower the orientation tensor develops with the flow, and the broader the core layer. For κ=1, the RSC model reduces to the Advani and Tucker model.

### 2.2. Nonlinear Anisotropic Structural Model

The nonlinear anisotropic mechanical behavior of the short glass fiber reinforced PPS was modeled following the multiscale approach proposed by Kenik and coworkers [14]. Under mechanical loading, injection-molded short glass fiber reinforced thermoplastics show significant plasticity before fracture. Moreover, such a degree of plasticity strongly depends on fiber orientation [15]. Besides, as the reinforcing fibers are short, fracture occurs primarily by tearing the polymeric matrix without breaking the reinforcing fibers, but with some degree of fiber pull-out [16]. Therefore, the followed multiscale approach is based on the following simplifying assumptions:

1. the reinforcing fibers exhibit a linear elastic response without any fracture;
2. the polymeric matrix exhibits both plasticity and fracture;
3. the matrix plasticity and fracture account for any fiber debonding;
4. all nonlinearity exhibited by the composite material is due to the polymeric matrix nonlinearity;
5. plasticity and fracture of the polymeric matrix are driven by stress in the matrix instead of homogenized stress in the composite;
6. the plasticity and fracture of the polymeric matrix strongly depend on the orientation of the reinforcing fibers, and this dependence increases with the degree of fiber alignment.

In a structural finite element simulation, the part deformation is based on the stiffness of the homogenized composite material. In the homogenization process of the multiscale material model, the properties of the matrix and the fibers are inputted into an incremental Mori–Tanaka micromechanical model, which generates homogenized properties for an ideal composite having perfectly aligned fibers [17]. These properties are then modified according to the fiber orientation tensor to produce the homogenized composite properties for the actual FOD.

However, to predict plasticity and rupture of the matrix material, the homogenized composite strain increments must be decomposed into the average strain increments in the polymeric matrix. The decomposition process is based on the tangent properties of the matrix and the fibers, the incremental Mori–Tanaka micromechanics model, and the fiber orientation tensor, as described in Nghiep et al. [18]. The calculated average strain increment for the polymeric matrix is inputted into the matrix plasticity model, which is described by the Ramberg–Osgood (RO) plasticity Equation [19]:

(12) $$\sigma_{Y}^{h}=E^{1/n}\sigma_{0}^{\left(n-1\right)/n}\epsilon_{p,eff}^{1/n},$$

where $\sigma_{Y}^{h}$ is the effective hardened yield strength, ***E*** is the elastic modulus, $\sigma_{o}$ and ***n*** are the yield strength and the hardening exponent used in the isotropic Ramberg–Osgood plasticity model, and $\epsilon_{p,eff}$ is the effective plastic strain in the polymeric matrix. The yield function is satisfied when the effective stress in the polymeric matrix equals the hardened yield strength expressed by Equation (12).

In order to consider the directional dependency of the matrix plasticity, the von Mises expression for the effective stress is modified as follows:

(13) $$\sigma_{eff}=\sqrt{\frac{{\left(\alpha \sigma_{11}-\beta \sigma_{22}\right)}^{2}+{\left(\beta \sigma_{22}-\beta \sigma_{33}\right)}^{2}+{\left(\beta \sigma_{33}-\alpha \sigma_{11}\right)}^{2}+6\left(\sigma_{12}^{2}+\sigma_{23}^{2}+\sigma_{31}^{2}\right)}{2}},$$

where the directionally dependent weighting coefficients (*α*, *β*) are linear functions of the degree of fiber alignment, which is quantified by the largest eigenvalue, $\lambda_{I}$, of the fiber orientation tensor:

(14) $$\alpha =\theta +\left(\frac{\alpha_{m}-\theta }{\lambda_{m,I}-1/2}\right)\left(\lambda_{I}-1/2\right),$$

(15) $$\beta =\theta +\left(\frac{\beta_{m}-\theta }{\lambda_{m,I}-1/2}\right)\left(\lambda_{I}-1/2\right),$$

In Equations (14) and (15), $\alpha_{m}$ and $\beta_{m}$ are the values of *α* and *β*, respectively, that are determined by fitting the response of a composite having highly aligned fibers, with a largest fiber orientation eigenvalue of $\lambda_{m,I}$. ***θ*** is the value that both *α* and *β* assume for a random fiber orientation.

In this model, the fracture criterion, which identifies the complete failure of the short fiber reinforced thermoplastic, is defined as an upper limit on the value of the modified effective stress (i.e., the effective strength, *S_eff_*):

(16) $$S_{eff}=\sqrt{\frac{{\left(\alpha \sigma_{11}-\beta \sigma_{22}\right)}^{2}+{\left(\beta \sigma_{22}-\beta \sigma_{33}\right)}^{2}+{\left(\beta \sigma_{33}-\alpha \sigma_{11}\right)}^{2}+6\left(\sigma_{12}^{2}+\sigma_{23}^{2}+\sigma_{31}^{2}\right)}{2}},$$

## 3. Materials and Methods

### 3.1. Material and Plate Design

A polyphenylene sulfide reinforced with 40 wt.% short glass fibers (PPS, Ryton R4 200 NA, Brussels, Belgium) was used in this work. Such material is considered a high-performance compound due to its high mechanical behavior in corrosive and high-temperature environments, and it is used for structural parts, especially in automotive applications. A titanate coupling agent is used in this compound to increase resin crystallization temperature, decrease isothermal crystallization, increase composite elongation at break, and eliminate embrittlement [20].

**Figure 1:**
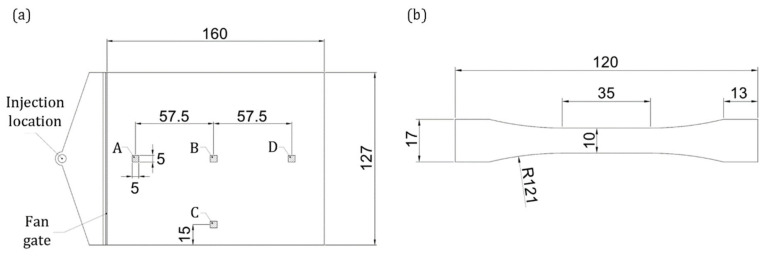
(**a**) Injection-molded plates and location of the investigated areas, and (**b**) cut-out tensile specimen design.

Several plates were injection molded to cut out tensile specimens with different fiber orientations. Since narrow gates significantly influence the orientation of the fibers within the cavity, a fan gate was used according to the ISO 294-3:2020 standard. The overall size of the plate was 127 × 160 × 1.8 mm^3^ (Figure 1a).

The evaluation of the fiber orientation was performed on μCT reconstructions of four regions of interest (ROI), as explained in Section 3.3. In order to study the FOD dependence on the shear strain, three ROIs (A, B, and D in Figure 1a) were located centrally and progressively further from the gate. The ROI C was located sideways to investigate edge influence. The ROIs’ dimensions were set at 5 × 5 × 1.8 mm^3^ to allow for high-resolution μCT scans. Figure 1b shows the design of the tensile specimens that were cut out from the plates at three different angles to the flow direction: 0°, 45°, and 90°.

### 3.2. Numerical Simulation of the Plate Molding

The simulations were performed with the software application Autodesk Moldflow Insight 2019 (AMI) using the RSC model and varying the values of the two fitting coefficients: *C_I_* and *κ*. The prediction performance was evaluated by comparing the error of the simulations conducted with the optimized and default coefficients, respectively. AMI defines the default values as *C_I_* = 0.05 and *κ* = 0.002.

A 3D mesh was created using 1.4 million tetrahedral elements for the plate model, with four nodes each. The model thickness was subdivided into 14 layers to simulate the FOD with an acceptable approximation. The process parameters used in the molding of the physical plates were used to set the initial and boundary conditions for the simulations. Moldflow laboratories provided an accurate rheological and thermal characterization of the PPS used.

### 3.3. FOD Measurements

The fiber orientation distribution was determined by scanning the selected ROIs with an industrial μCT system (Nikon Metrology MCT225, Leuven, Belgium), characterized by a 225 kV micro-focus X-ray source (minimum focal spot size equal to 3 µm), a 2000 × 2000 pixels flat-panel detector (16 bit) and a temperature-controlled cabinet. The scans were performed with a voxel size of 3 µm. A detailed description of the scanning and reconstruction procedures is provided in a previous work [21].

The obtained µCT three-dimensional reconstructions of the four ROIs of the plate were analyzed using the software VGStudio MAX 3.2 (Volume Graphics GmbH, Heidelberg, Germany), which allowed the direct extraction of the components of the fiber orientation tensor (*T_xx_*, *T_yy_*, and *T_zz_*) through the thickness of the plate.

### 3.4. Optimization of the FOD Prediction

The identification of the RSC model coefficients (*C_I_*, *κ*) was conducted using a Genetic Algorithm and an Artificial Neural Network as a machine learning-based surrogate model to reduce the computational time [22]. The ANN was trained using the input and output of the AMI simulation. The ranges for the model coefficients were set according to Moldflow recommendations as [23]:

(17) $$C_{I}\in \left[0\;,\;0.1\right],\;\kappa \in \left[0.0001\;,\;1\right],$$

In order to cover the whole domain, a five-level full factorial was used, i.e., 25 uniformly distributed coefficients pairs were defined. After a preliminary comparison between the experimental and numerical results, an additional level was added for the coefficient *C_I_*, to better cover the optimum location range.

The ANN construction and training were performed using Matlab R2018b [24]. The input and output data were divided as follows: 70% was used for training, 15% for validation, and 15% for testing.

Four different ANNs were used for the optimization process: one for each analyzed ROI. For each ANN, the number of neurons in the hidden layer was selected considering a trade-off between the time needed for training and the ANN ability to fit the output data. The ANNs were trained to reproduce the simulation results for all the 25 pairs of RSC coefficients using Levenberg–Marquardt backpropagation [25].

The calculated FOD was then compared with the experimental one for each ROI and each component of the fiber orientation tensor. The absolute value of the difference vector between the numerical and experimental data was minimized using a MOGA-II genetic optimization algorithm [26]. MOGA-II was selected for its robustness and fast convergence, as it uses an intelligent multi-search elitism and directional crossover. It uses four different operators for reproduction: mutation, selection, classical crossover, and directional crossover. One of the four operators is chosen at each step of the reproduction process and applied to the current individual [26]. The previously trained ANNs approximated the numerically calculated FOD for each ROI and any RSC coefficient pairs to compare them with the relevant μCT scan results.

### 3.5. Identification of the Ramberg–Osgood Model Parameters

The tensile tests on the cut-out specimens were performed using an MTS Minibionix servo-hydraulic test machine equipped with a 15 kN load cell, with a deformation velocity of 1 mm/min. An MTS extensometer with a gauge length of 35 mm was used to evaluate the strain. The Ramberg–Osgood multiscale structural model described in Section 2.2 was fitted to the results of the tensile tests in two alternative approaches: curve fitting and inverse modeling.

The curve fitting approach was conducted following two steps:

1. The elastic modulus and Poisson ratio values for the polymeric matrix and the glass fibers were determined by requiring the model to accurately fit the initial elastic responses of the 0°, 90°, and 45° tensile test specimens.
2. The four plastic coefficients, *σ_0_*, *n*, *α*, and *β*, and the effective strength, *S_eff_*, were then determined by fitting the model to the complete stress-strain curve for all three tensile tests.

In the inverse modeling approach, the fiber orientation results obtained from the AMI simulations were translated into local mechanical properties using the software application Autodesk Helius Advanced Material Exchange (AME) [27]. A numerical simulation of the tensile test was set up for each of the three considered orientations using the software application ANSYS Mechanical APDL. For each element of the AMI mesh, the predicted FOD was mapped into the corresponding element in the structural model with the relative strength characteristics. The load was divided into 70 substeps to evaluate the rupture time and the behavior until the rupture. Eventually, the stress-strain curves were calculated using the tensile test simulations and compared with the experimental results. The Ramberg–Osgood model parameters were then identified by inverse modeling, i.e., varying them until the calculated stress-strain curves fitted the experimental curves, minimizing the deviation between them [28]. The inverse modeling was conducted using a Genetic Algorithm and an Artificial Neural Network as a machine learning-based surrogate model to reduce the computational time, as explained in Section 3.4.

### 3.6. Validation

**Figure 2:**
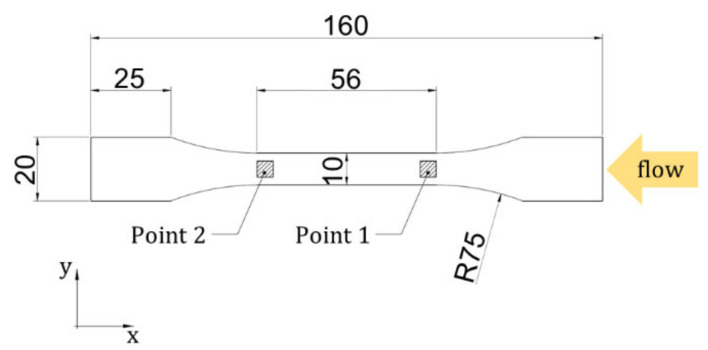
Design of the injection-molded tensile specimen (thickness: 4 mm).

The proposed approach was validated using the experimental stress-strain curve of a different tensile specimen, directly injection-molded in a dumbbell-shaped cavity. Figure 2 shows the design of the tensile specimen that was molded using the same compound and the same process conditions adopted for the plate. Unlike the plate, the convergent geometry of the cavity and its narrower section promotes a higher fiber orientation in the flow direction. Fiber orientation was evaluated by µCT scans in two ROIs, having the same dimensions of the ROIs analyzed on the plate: Point 1 and Point 2 in Figure 2.

The process simulation was performed using AMI, using a 3D mesh of 1 million tetrahedral elements, with four nodes each. The model thickness was subdivided into 14 layers to simulate the FOD with an acceptable approximation. The process parameters used in the molding of the actual tensile specimen were used to set the initial and boundary conditions for the simulation. Using the software tool AME, the fiber orientation results of the AMI simulations were translated into local mechanical properties [27]. ANSYS Mechanical APDL was used to numerically simulate a tensile test by constraining the nodes at one end of the specimen and applying a displacement of 1.5 mm to the other end. The calculated FOD was mapped into the corresponding element in the structural model, together with the relative strength characteristics, for each element of the AMI mesh. To analyze the rupture time and behavior until the rupture, the load was divided into 70 substeps. The stress-strain curves were then examined and compared with the tensile test outcomes.

## 4. Results and Discussion

### 4.1. Optimization of the RSC Model Coefficients

**Figure 3:**
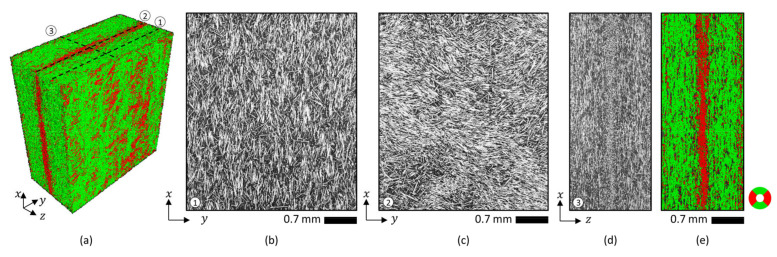
(**a**) Example of three-dimensional µCT reconstruction of a molded plate’s region (ROI A). Examples of bi-directional cross-sections extracted from the µCT reconstruction at different positions represented with white dashed lines: (**b**) position 1, (**c**) position 2, and (**d**,**e**) position 3. Fiber orientation analysis (two-color map showing orientation angles according to the angular scale) is shown both in (**a**) 3D and (**e**) 2D.

Figure 3 shows as an example the µCT three-dimensional reconstruction and the fiber orientation analysis of ROI A, together with extracted bi-dimensional cross-sections where glass fibers and polymer matrix are represented by different gray value ranges (bright and dark, respectively).

**Figure 4:**
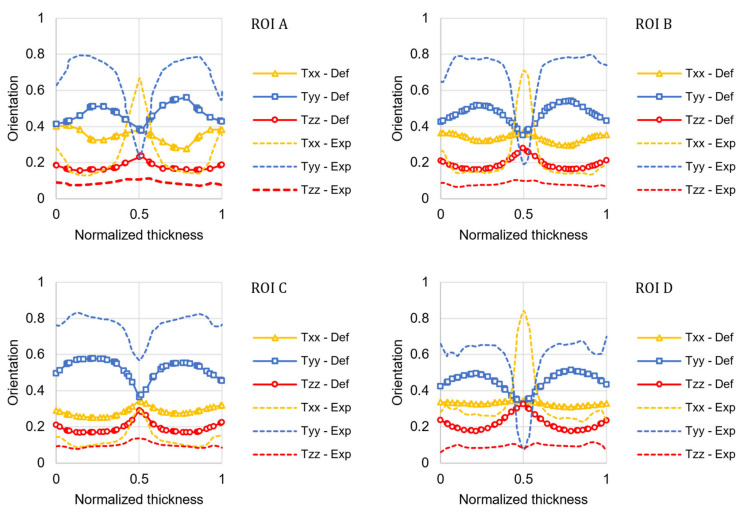
Comparison between the experimental results (Exp) of the fiber orientation tensor principal components and the numerical results predicted using the default coefficients of the RSC model (Def) for the four ROIs in the plate.

Figure 4 shows the comparison of FOD obtained from µCT experimental results and the numerical results obtained using AMI with the default coefficients of the RSC model (*C_I_* = 0.05 and *κ* = 0.002). *T_zz_* represents the orientation in the through-thickness direction, *T_xx_* the orientation transverse to the flow direction, and *T_yy_* the alignment of the fibers in the flow direction.

In all the four ROIs, the fiber orientation in the flow direction (*T_yy_*) varies as expected: due to the fountain flow, the fibers are randomly oriented in the core layer and highly oriented in the shear layer near the surface [29]. Most of the fibers are oriented in the flow and transverse direction, with only a small amount of fibers oriented in the thickness direction. The FOD of the ROIs located along the plate axis (A, B, and D) is similar, whereas in ROI C (which is located to the side), the fibers in the core are more oriented in the flow direction due to the border effect.

**Figure 5:**
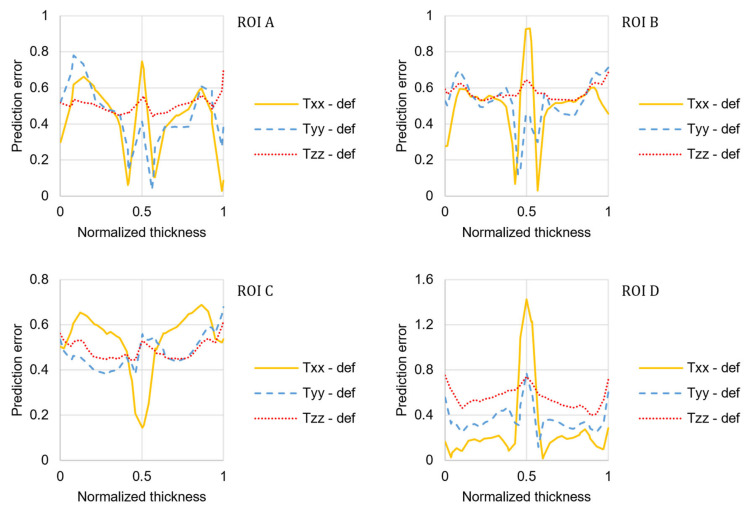
Predictions error distribution obtained with the default coefficients of the RSC model.

It can be observed that the RSC model with the default coefficients roughly resembles the experimental FOD in shape, but it fails to predict its magnitude. The relative prediction error was calculated for each component of the orientation tensor as follows and reported in Figure 5:

(18) $$\varepsilon_{r}=\frac{\left|T_{rr,num}-T_{rr,exp}\right|}{T_{rr,exp}},$$

where *T_rr_* is *T_xx_*, *T_yy_* or *T_zz_* evaluated for each location along the thickness direction.

**Figure 6:**
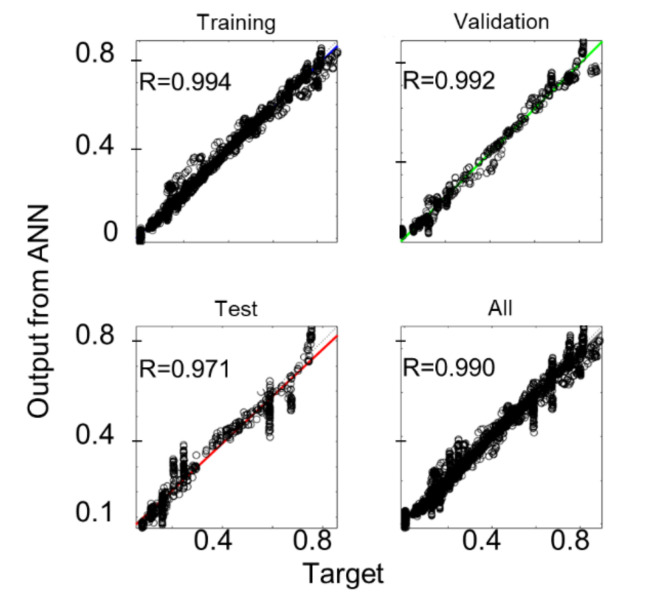
Results of the ANN training for ROI A.

The application of the proposed optimization algorithm allowed the identification of the optimal values for the RSC model coefficient: *C_I_* = 0.0138 and *κ* = 0.2056. Figure 6 shows the results of the ANN training for the ROI A. The numerical simulation results are reported in the *x*-axis, and the output calculated by the ANN is on the *y*-axis.

Here, *R* is the angular coefficient of the linear regression: the closer *R* is to 1, the more accurate the ANN is in approximating the numerical simulation FOD prediction.

**Figure 7:**
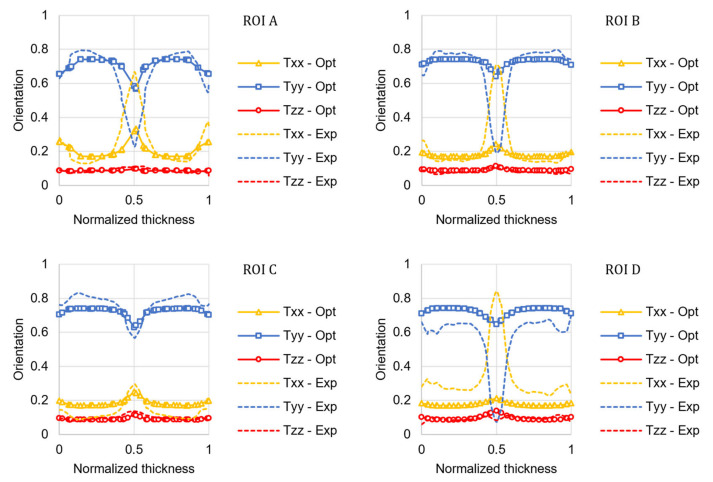
Comparison between the µCT experimental results (Exp) of the fiber orientation tensor principal components and the numerical results predicted using the optimized coefficients (Opt) of the RSC model for the four ROIs in the plate.

The numerical simulation conducted using the optimized coefficients significantly improved the predicted orientation accuracy, as illustrated in Figure 7.

**Figure 8:**
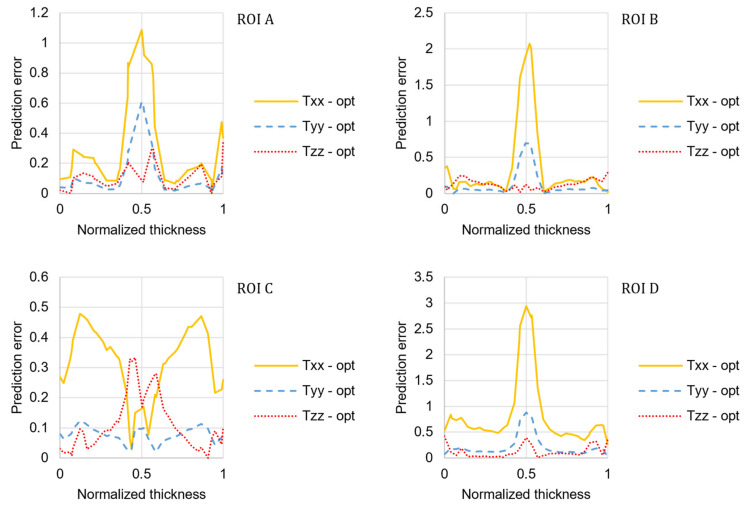
Error distribution for the predictions obtained with the optimized coefficients of the RSC model.

The fiber orientation prediction in the shear layers is accurate in both distribution shape and magnitude. For the ROIs located along the plate axis (A, B, and D), the model cannot predict the high transverse orientation that characterizes the core layer. In the core layer, the fiber orientation is under-predicted, as shown by Kleindel and coworkers for parts with chunky geometry (i.e., cross-section of 5 × 5 mm^2^) [30]. As elongation flows are dominant in the core layer, a possible explanation for this underprediction is that the coefficients of the fiber orientation model were fitted for a plate that is mainly filled in pure shear, and they cannot be transferred to other flow regimes [6]. The relative prediction error for the optimized model was calculated for each component of the orientation tensor and reported in Figure 8.

**Figure 9:**
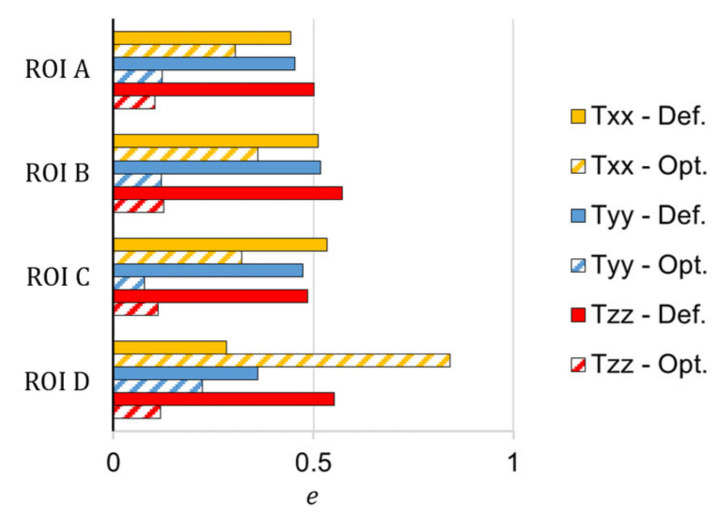
The discrepancy between the measured and predicted fiber orientations.

The following discrepancy expression was used to evaluate the overall deviation between the measured fiber orientation (as a second-order tensor) and the predicted results (with the default and optimized RSC model coefficients, respectively) across the thickness [12]:

(19) $$e=\frac{\int ∥T^{\left(num\right)}-T^{\left(exp\right)}∥dz}{\int ∥T^{\left(exp\right)}∥dz},\;∥T∥=\sum_{\left(i,j\right)\in \Gamma }\left|T_{ij}\right|,$$

where *z* represents the position across the thickness or flow path distance, and *Γ* denotes the fiber orientation components used for error computation. Figure 9 shows the discrepancy between the measured fiber orientation and the predicted results with the default and optimized RSC model coefficients, respectively, and for each ROI of the plate.

The optimized coefficients significantly improved the prediction accuracy for all the ROIs and the fiber orientation components, except for the orientation transverse to the flow direction that is overpredicted for both the core and the shear layers in ROI D.

Overall, the proposed optimization approach, based on a MOGA-II GA and an ANN as a machine learning-based surrogate model, reduced the FOD prediction error by 51%, which is a substantial improvement compared with the gradient-based algorithms previously used in the literature [11,12].

### 4.2. Properties Prediction for the Injection-Molded Tensile Specimen

**Figure 10:**
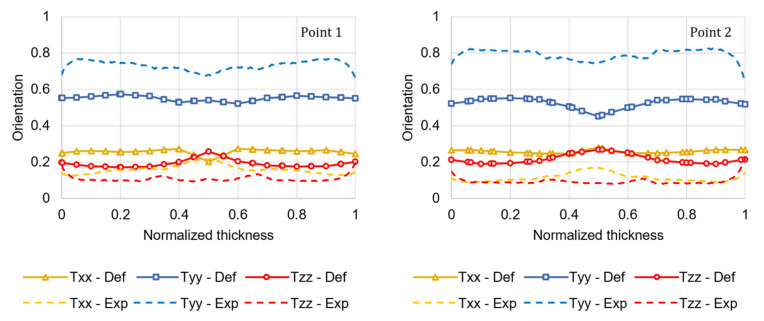
Comparison between the µCT experimental results (Exp) of the fiber orientation tensor principal components and the numerical results (Def) predicted using the default coefficients of the RSC model for the two ROIs in the tensile specimen.

Figure 10 shows the FOD comparison between the experimental results and the numerical results obtained for the injection-molded tensile specimen introduced in Section 3.6 with the default coefficients of the RSC model (*C_I_* = 0.05 and *κ* = 0.002). The high orientation in the flow direction is due to the combined effect of the side walls proximity and the elongational flow caused by the cross-section reduction.

**Figure 11:**
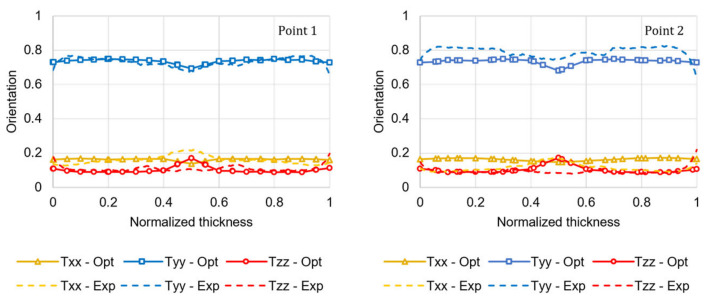
Comparison between the µCT experimental results (Exp) of the fiber orientation tensor principal components and the numerical results (Opt) predicted using the optimized coefficients of the RSC model for the two ROIs in the tensile specimen.

The numerical simulation conducted using the optimized coefficients (*C_I_* = 0.0138 and *κ* = 0.2056) significantly improved the predicted orientation accuracy, as illustrated in Figure 11. The FOD prediction error, calculated using Equation (19) for both ROIs, is reduced by 77%. Compared with the prediction accuracy of the FOD of the plate, this better performance is due to the very thin core layer that characterizes the injection-molded tensile specimen. As mentioned in Section 4.1, the RSC model does not accurately predict the high transverse orientation that characterizes the core layer.

**Figure 12:**
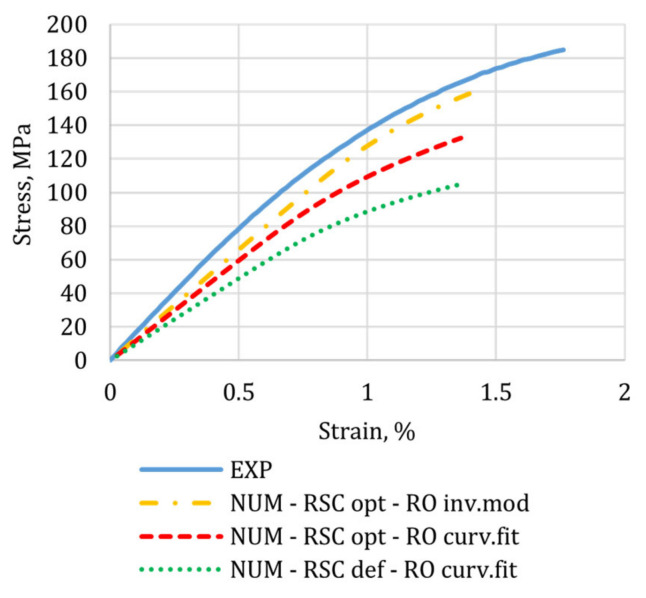
Numerical and experimental stress-strain curves for the injection-molded tensile specimen.

The optimized FOD prediction was then translated into local mechanical properties to run a structural simulation of the tensile test. Figure 12 compares the injection-molded tensile specimen’s numerical and experimental stress-strain curves.

As expected, the higher degree of fiber alignment along the load direction, predicted using the optimized RSC coefficients (and the curve-fitted Ramberg–Osgood parameters), increased the composite modulus and strength, reducing the discrepancy from the experimental curve. As reported in Table 1, the prediction accuracy improved by 30% (67–37%) and 35% (75–40%) for the composite’s elastic modulus and tensile strength, respectively. However, the experimental results are still largely underestimated. This is partly due to the RSC model’s difficulties in modeling the *T_yy_* variation along the flow path, as evident in Figure 10 (Point 2) and Figure 7 (D).

The proposed data-driven approach, which calculates both the RSC coefficients and the Ramberg–Osgood parameters by inverse modeling from experimental data, allowed for improvements in the prediction accuracy by 43% (67–24%) for the elastic modulus and 59% (75–16%) for the tensile strength, respectively.

## 5. Conclusions

Overall, the proposed optimization approach, based on a GA and an ANN, reduced the FOD prediction error by 51%, which is a substantial improvement compared with the gradient-based algorithms previously used in the literature.

The optimized FOD predictions were then translated into local mechanical properties, using tensile specimens cut out from the plate at different angles. The RO model parameters were identified using two alternative approaches: curve fitting and inverse modeling. They were then used to simulate a tensile test for a specimen injection molded in a dumbbell-shaped cavity selected as a case study for validation.

Numerical and experimental results (both μCT scans and mechanical characterization of the validation specimen) were finally compared to assess the impact of the proposed data-driven approach on the accuracy of the mechanical behavior prediction.

Using the RSC optimized coefficients, the FOD prediction error was reduced by 77%. Compared with the prediction accuracy of the FOD of the plate, this better performance is due to the very thin core layer that characterizes the injection-molded tensile specimen. The proposed data-driven approach, which calculates both the RSC coefficients and the RO parameters by inverse modeling from experimental data, improved the prediction accuracy by 43% for the elastic modulus and 59% for the tensile strength, respectively.

The results obtained for this polyphenylene sulfide reinforced with 40 wt.% short glass fibers (PPS, Solvay, Ryton R4 200 NA) are case-specific and cannot be generalized to other materials. However, the same approach can be applied to any other system to improve the prediction accuracy by tailoring the RSC coefficients and the RO parameters through inverse modeling from experimental data.

The proposed approach is not limited to the RSC and RO models, but it can be applied in the future to other advanced fiber orientation and mechanical models with a larger number of parameters. It is suggested that the systematic and robust optimization technique provided here can identify the optimal fiber orientation and mechanical parameters throughout the molding process when combined with a well-designed process and the accompanying flow kinematics describing injection molding.

## Figures and Tables

**Table 1:** Measured and predicted mechanical properties.

	Values		Error	
	E, MPa	Stress at Failure, MPa	E, %	Stress at Failure, %
Experimental values	16,414	185	-	-
Predicted with default RSC coefficients and curve-fitted RO parameters	9822	106	67%	75%
Predicted with optimized coefficients and curve-fitted RO parameters	11,972	132	37%	40%
Predicted with optimized RSC coefficients and inverse modeling from experimental data	13,256	160	24%	16%
